# Evolution of Human Brain Left–Right Asymmetry: Old Genes with New Functions

**DOI:** 10.1093/molbev/msad181

**Published:** 2023-08-10

**Authors:** Jianguo Wang, Sidi Ma, Peijie Yu, Xionglei He

**Affiliations:** State Key Laboratory of Biocontrol, School of Life Sciences, Sun Yat-sen University, Guangzhou, Guangdong Province, China; State Key Laboratory of Biocontrol, School of Life Sciences, Sun Yat-sen University, Guangzhou, Guangdong Province, China; State Key Laboratory of Biocontrol, School of Life Sciences, Sun Yat-sen University, Guangzhou, Guangdong Province, China

**Keywords:** brain asymmetry, human brain, gene age, left–right asymmetry, brain evolution, brain gene expression

## Abstract

The human brain is generally anatomically symmetrical, boasting mirror-like brain regions in the left and right hemispheres. Despite this symmetry, fine-scale structural asymmetries are prevalent and are believed to be responsible for distinct functional divisions within the brain. Prior studies propose that these asymmetric structures are predominantly primate specific or even unique to humans, suggesting that the genes contributing to the structural asymmetry of the human brain might have evolved recently. In our study, we identified approximately 1,500 traits associated with human brain asymmetry by collecting paired brain magnetic resonance imaging features from the UK Biobank. Each trait is measured in a specific region of one hemisphere and mirrored in the corresponding region of the other hemisphere. Conducting genome-wide association studies on these traits, we identified over 1,000 quantitative trait loci. Around these index single nucleotide polymorphisms, we found approximately 200 genes that are enriched in brain-related Gene Ontology terms and are predominantly upregulated in brain tissues. Interestingly, most of these genes are evolutionarily old, originating just prior to the emergence of Bilateria (bilaterally symmetrical animals) and Euteleostomi (bony vertebrates with a brain), at a significantly higher ratio than expected. Further analyses of these genes reveal a brain-specific upregulation in humans relative to other mammalian species. This suggests that the structural asymmetry of the human brain has been shaped by evolutionarily ancient genes that have assumed new functions over time.

## Introduction

While the human brain largely displays bilateral symmetry, with one-to-one corresponding regions in the left and right hemispheres, it also showcases notable structural asymmetry within these corresponding regions. This asymmetry underpins functional dominance in one hemisphere, a phenomenon referred to as brain lateralization or hemispheric specialization (Duboc et al. 2015; Kong et al. 2018; Sha, Schijven, et al. 2021; Saltoun et al. 2023; Williams et al. 2023). Brain lateralization plays a crucial role in various advanced neural functions, such as consciousness (Hartwigsen et al. 2021), speech (Poeppel and Assaneo 2020), language (Eckert et al. 2022), memory (Bein et al. 2020), cognitive performance (Wu et al. 2022), and handedness (Sha, Pepe, et al. 2021), among others. For instance, unique thickness asymmetries in the postcentral gyrus and inferior occipital cortex have been associated with left-handedness (Sha, Pepe, et al. 2021). Additionally, abnormalities in brain asymmetry have been linked to various neuropsychiatric disorders (Cardinale et al. 2013; Li et al. 2020; Sha, Schijven and Francks 2021; Park et al. 2022; Schijven et al. 2023).

An intriguing facet is that brain asymmetry in humans appears to be of recent origin, given that many human brain asymmetry features are either primate-specific or human-specific (Leroy et al. 2015; Marie et al. 2018; Graic et al. 2020; Neubauer et al. 2020; Xiang et al. 2020; Gonzalez et al. 2022; Wan et al. 2022; Hill et al. 2023). For instance, the human brain presents a similar but more variable spatial asymmetry pattern compared with great apes (Neubauer et al. 2020), and there are substantial asymmetry differences between human and chimpanzee in terms of surface area and cortical thickness (Xiang et al. 2020). Furthermore, certain brain regions and structures demonstrate unique asymmetries rarely seen in other species (Leroy et al. 2015; Hill et al. 2023). Some brain asymmetry patterns, however, are shared with other primates (Marie et al. 2018; Gonzalez et al. 2022). Mouse brains, when compared, display asymmetry primarily in hippocampus size, with shape asymmetry observed in most examined regions, but insignificant volume asymmetry (Spring et al. 2010; Parnell et al. 2013; Barbeito-Andres et al. 2016). Hence, most, if not all, observed brain asymmetries in human brain appear to be evolved recently.

In this research, we explore the evolutionary origins of the genes implicated in human brain asymmetry. Aspects of human brain evolution, such as cortical expansion (Won et al. 2019), are primarily driven by changes in gene regulation, with additional contributions from recently evolved genes like *SRGAP2C*, a product of human-specific gene duplication that notably enhances corticocortical connectivity (Vanderhaeghen and Polleux 2023). The pivotal question we aim to resolve is whether the genes underpinning human brain asymmetry are recent developments or if they are ancient genes that have acquired new functions over time. To address this, we scrutinized variations in brain asymmetry features in approximately 30,000 individuals from the UK Biobank (Bycroft et al. 2018), intending to unveil the underlying genes. Earlier studies have begun to illuminate the genetic foundations of specific brain asymmetry features. For instance, one research group identified 27 lead single nucleotide polymorphisms (SNPs) after examining 73 traits linked to brain asymmetry, focusing particularly on cortical thickness, surface area, and subcortical volume (Sha, Schijven, et al. 2021). Another study investigated asymmetry in brain torque components, discovering 86 lead SNPs (Zhao et al. 2022). Nevertheless, these studies predominantly targeted specific types of brain asymmetry features.

Our study adopted a comprehensive approach. We broadened the scope to encompass a diverse range of 1,504 brain asymmetry traits, including both white and gray matter, and various magnetic resonance imaging (MRI) features across around 100 brain regions. This enabled a more holistic perspective of brain structural asymmetry. As a result, we pinpointed 1,195 quantitative trait loci (QTLs) accounting for 337 of the 1,504 brain asymmetry traits. We also located 216 genes in proximity to the index SNPs. Functional analysis revealed significant enrichment of these brain asymmetry genes in brain-related Gene Ontology (GO) terms. Furthermore, these genes exhibited significant enrichments in upregulated differentially expressed genes (DEGs) in brain tissues. To investigate the evolutionary origins of these genes, we performed a macroevolutionary analysis based on clade-specific gene origins (Capra et al. 2012; Shao et al. 2019). Intriguingly, these genes appear to have significantly emerged just prior to the advent of Bilateria (bilaterally symmetrical animals) and Euteleostomi (bony vertebrates with a brain), both ancient clades. These genes also showed marked upregulation in the human brain compared with other species. In conclusion, our findings suggest that genes contributing to brain asymmetry originated in ancient times but have since evolved to perform new functions.

## Results

### Definition of Left–Right Brain Asymmetry Traits

**Fig. 1.**
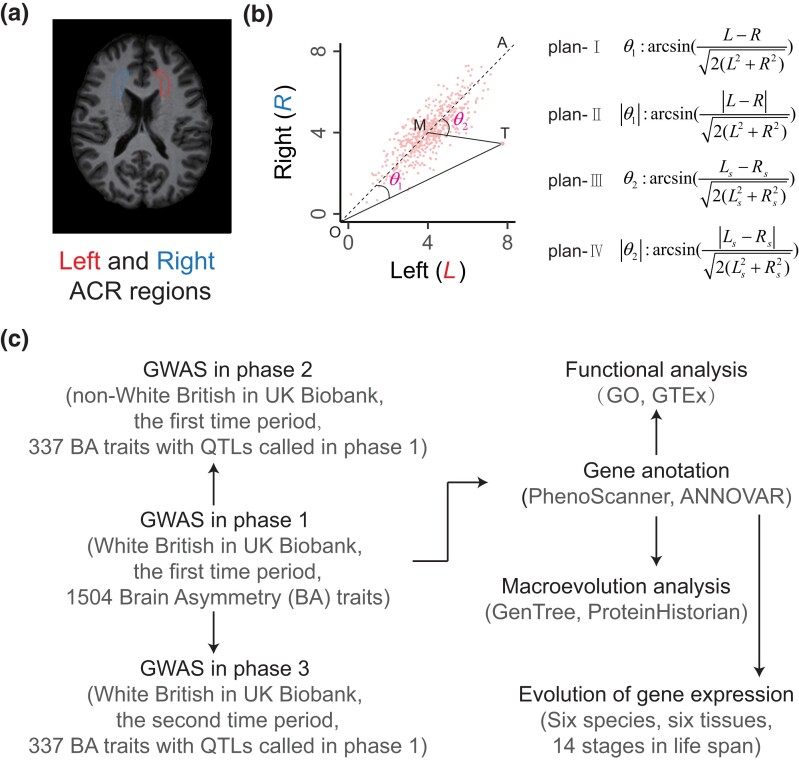
Defining brain asymmetry traits. (*a*) An illustration of a pair of traits in a left brain region and a corresponding right brain region, with ACR representing anterior corona radiata. (*b*) The portrayal of brain asymmetry traits according to four different plans. Point M represents the population averages for trait pair (*L* and *R*). Point T represents observed trait values for an individual’s trait pair. Point O denotes the origin. The dashed line OA represents *L* = *R*, with *L*_s_ and *R*_s_ being the standardized values of *L* and *R*, respectively. (*c*) The overall analytical framework of this study.

We gathered human brain traits captured via MRI from the UK Biobank (Bycroft et al. 2018). These traits were collected from approximately 30,000 genotyped British individuals representing various ancestries. Among the collected traits, 376 pairs correspond to measurements of symmetrical brain regions. The set of 376 trait pairs includes all available brain traits obtained during the download process, where one trait is measured in a left-brain region and the corresponding trait is measured in the corresponding right-brain region (fig. 1*a*). These traits encompass around 100 brain regions, such as the accumbens and acoustic radiation, and encompass multiple features. These features consist of 72 pairs related to volumes of white and gray matter, as well as 297 pairs pertaining to white matter structure ascertained through diffusion MRI. Moreover, for each pair of traits, we considered two distinct time periods (see Materials and Methods).

For each pair of traits (*L* and *R*), we defined asymmetry traits corresponding to the trait pair by $arcsin((L-R)/\sqrt{2(L^{2}+R^{2})})$ or $arcsin((L_{s}-R_{s})/\sqrt{2(L_{s}^{2}+R_{s}^{2})})$ as well as their absolute form, where *L*_s_ and *R*_s_ represent the standardized trait values of *L* and *R*, respectively (fig. 1*b*). Consequently, we procured 1,504 (376 × 4) brain left–right asymmetry traits measured at two time periods (supplementary table S1, Supplementary Material online). The analysis pipeline is displayed in figure 1*c*. The traditional formula ((*L* − *R*)/(*L* + *R*)) (Sha, Schijven, et al. 2021) excels with exclusively positive trait values but struggles when values span from positive to negative, failing to consider their distribution. In response, we have refined this formula to deepen our understanding of brain asymmetry and improve the detection of underlying genes. Our updated definitions match the traditional formula for positive *L* and *R*, and normalized derived asymmetry traits, but they uniquely consider trait value distribution. They also offer a clearer geometric interpretation, specifically the deviation angle from the line *L* = *R*. A thorough mathematical review of the advantages of our revised definitions over the traditional formula and their connections is detailed in the supplementary note, Supplementary Material online.

### Identification of Genetic Variants Associated with Brain Left–Right Asymmetry Traits

**Fig. 2.**
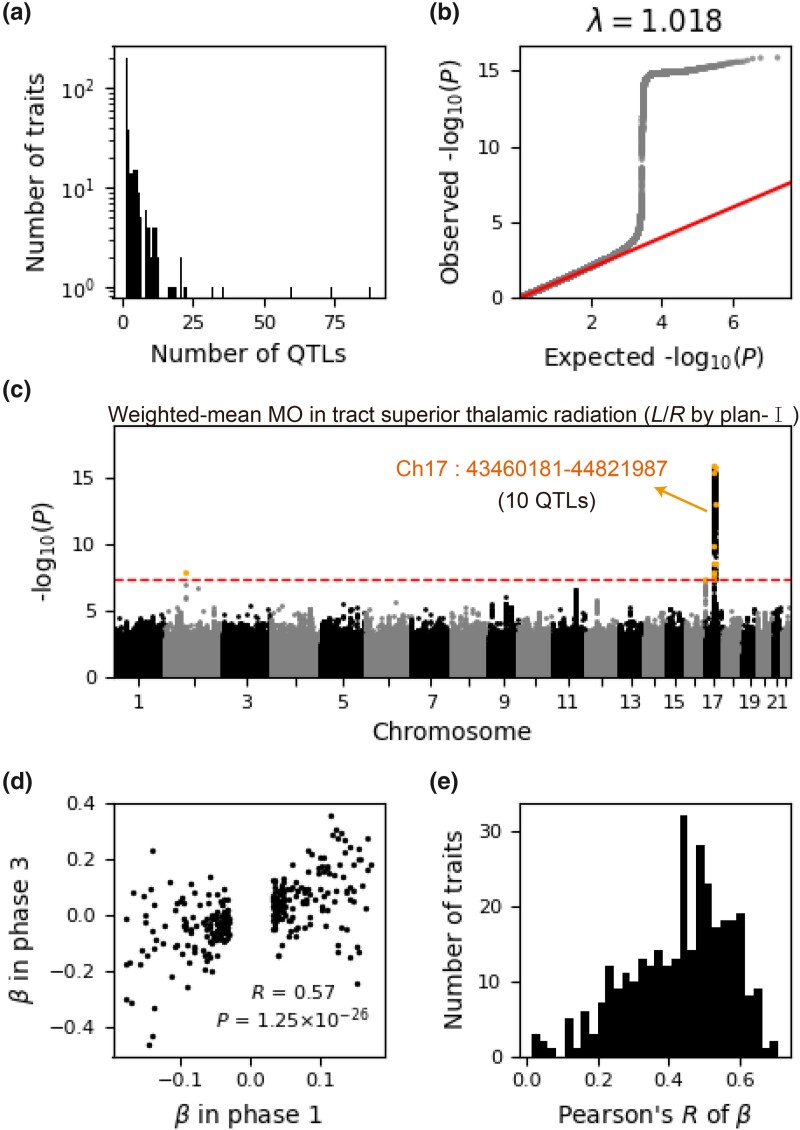
GWAS of brain asymmetry traits. (*a*) The distribution of QTL numbers for the 337 identified traits each having at least one QTL. The effect sizes and corresponding *P* values are depicted in supplementary figure S1, Supplementary Material online. (*b*) The Q–Q plot illustrates the well-controlled population structure for an example asymmetry trait defined by plan-I for the weighted-mean MO in the tract superior thalamic radiation of left and right hemispheres. MO stands for diffusion tensor mode. Red and gray colors represent expected and observed *P* values, respectively. The inflation factor *λ* = 1.018 is very close to 1. (*c*) The Manhattan plot for the trait used in (*b*), with ten QTLs positioned within the range Ch17: 43460181–44821987. The red dashed line marks the significance threshold *P* < 5 ×10^−8^, and the index SNPs corresponding to QTLs are highlighted in yellow. (*d*) The scatter plot illustrates a significant positive Pearson's correlation (*R*) between the *β* coefficients of phase 1 and phase 3 for the trait examined in (*b*). (*e*) The Pearson correlation (*R*) between the *β* coefficients of phase 1 and phase 3 for all of the 337 traits. The corresponding *P* values are depicted in supplementary figure S2, Supplementary Material online.

In our initial investigation, we conducted genome-wide association studies (GWAS) for each of the 1,504 brain asymmetry traits in the White British population during the first time period (phase 1). Each trait was normalized, SNPs were filtered to include 8,895,704, and GWAS was implemented through GCTA (Jiang et al. 2019). We estimated the genetic relatedness matrix (GRM) using SNPs overlapping with linkage disequilibrium (LD)-pruned HapMap3 SNPs and considered covariates including age, sex, age^2^, age × sex, and age^2^ × sex as well as the top 20 principal components, as outlined in a previous study (Jiang et al. 2019). Summary statistics for all 1,504 asymmetry traits were obtained, and at a significance level of *P* < 5 × 10^−8^, we discerned significant genetic variants, defining the QTLs via clumping analysis (Purcell et al. 2007) for each trait. Consequently, 1,195 QTLs were identified for 337 of the 1,504 traits (fig. 2*a*; supplementary table S2, Supplementary Material online; see Materials and Methods). The effect sizes, represented by the *β* coefficients of these QTLs, are displayed along with their respective *P* values in supplementary figure S1, Supplementary Material online. For instance, the asymmetry trait of plan-I, corresponding to weighted-mean MO (diffusion tensor mode) in the superior thalamic radiation tract, contained 12 QTLs. The Q–Q plot for this trait demonstrated effective control of the population structure (inflation factor *λ* = 1.018; fig. 2*b*), and the Manhattan plot exhibited the genome-wide distribution of significant genetic variants (fig. 2*c*). As the original brain MRI traits derive from identical MRI images for the same subjects, the population structure should be well controlled for other traits.

We then performed a replication analysis in British individuals of other ancestries (phase 2) (see Materials and Methods), procuring summary statistics for each of the 337 traits and testing the significance of QTLs identified after clumping in phase 1 at *P* < 0.05/*n* = 4.56 × 10^−5^, where *n* = 1,096 represents the count of QTLs shared in phase 2 (Makowski et al. 2022). A total of 19 QTLs remained significant in phase 2, a significant outcome based on a binomial test (*P* = 1.28 × 10^−42^). The sign concordance rate (SCR) of top significant SNPs between phases 1 and 2 was also statistically significant (SCR = 0.54, *P* = 8.65 × 10^−90^, binomial test) (see Materials and Methods) (Makowski et al. 2022).

In the subsequent replication analysis in the White British population measured at a second time period (phase 3) (see Materials and Methods), the number of QTLs remaining significant was also noteworthy (*n* = 1, *P* = 0.049, binomial test), as was the SCR between phases 1 and 3 (SCR = 0.69, *P* < 8.65 × 10^−90^, binomial test). We calculated Pearson’s *R* between the *β* coefficients of top significant SNPs in phases 1 and 2 or 3, finding a significant correlation for most traits between phases 1 and 3 (fig. 2*d* and *e*; supplementary fig. S2, Supplementary Material online), but not between phases 1 and 2 due to the smaller sample size and complex population structure in phase 2 (see Materials and Methods) (Makowski et al. 2022). These results from phases 2 and 3 underscore the robustness of the identified genetic variants against population structure and temporal variation.

#### Gene Annotation and Functional Enrichment

We began by associating the genes with each of the 1,195 index SNPs using two software tools: PhenoScanner (Kamat et al. 2019) and ANNOVAR (Wang et al. 2010) (see Materials and Methods). Noting a high degree of overlap in the genes annotated by these two tools (supplementary fig. S3, Supplementary Material online), we combined the annotations from both, resulting in 216 genes (supplementary table S3, Supplementary Material online). These are henceforth referred to as “brain asymmetry” (BA) genes. Notably, some of the BA genes we identified, such as *MAP2* and *MAPT* known for their involvement in brain asymmetry and neurodegenerative diseases (Lubben et al. 2021), have also been implicated in previous studies (supplementary table S3, Supplementary Material online).

**Fig. 3.**
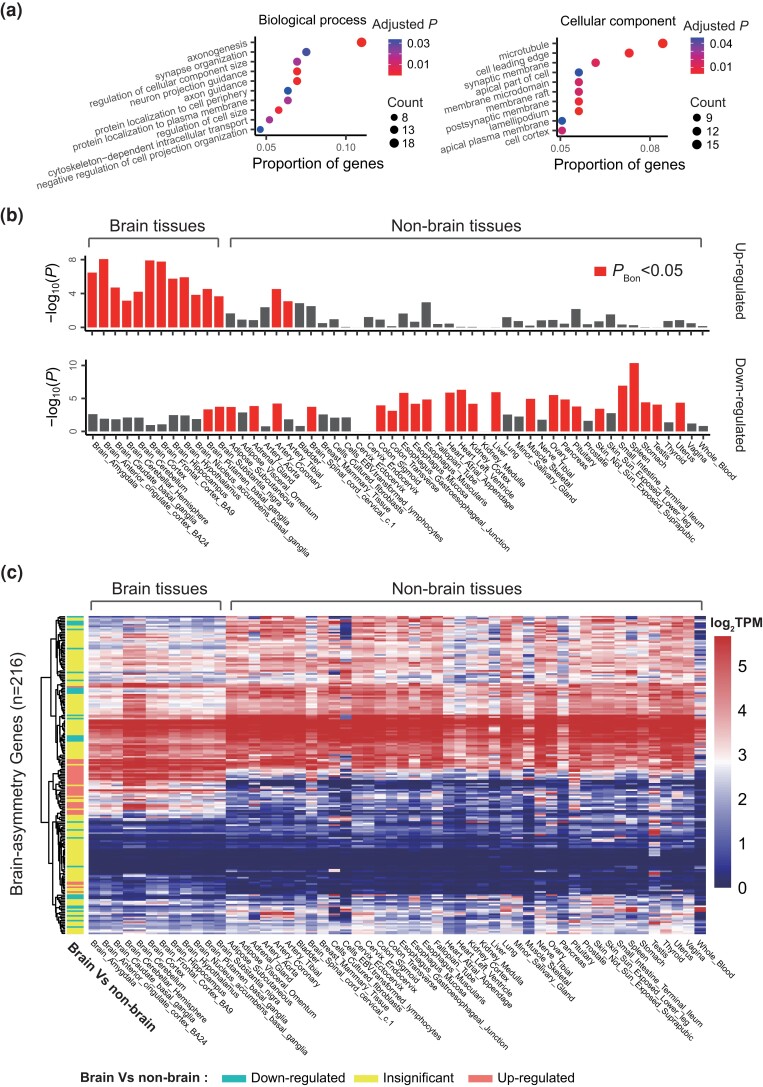
Functional enrichments of BA genes. (*a*) The displayed GO enrichments of BA genes involve biological processes and cellular components, derived using the R package “clusterProfiler.” The top 10 GO terms are presented, with the adjusted *P* value obtained through the hypergeometric test and adjusted by the Benjamini–Hochberg method. (*b*) The enrichments of BA genes in upregulated and downregulated DEGs of brain and nonbrain tissues are shown. The *P* value is obtained via a hypergeometric test. Red bars denote *P* < 0.05 post-Bonferroni correction. (*c*) The gene expression levels of BA genes in brain and nonbrain tissues are presented (from online FUMA using GTEx gene expression data). Each row represents one of the 200 BA genes, and each column denotes a tissue. The expression level in each cell is the average log_2_(Transcripts Per Million [TPM]) among replicates (as reported by online FUMA). For each gene, it is determined whether the gene expression levels in brain tissues are significantly upregulated or downregulated compared to nonbrain tissues using a *t*-test after Bonferroni correction (*P* < 0.05/*n*, where *n* is the number of genes). Notably, 16 genes are not included in the FUMA results.

Subsequently, we performed a GO analysis on these genes (see Materials and Methods) (Wu et al. 2021), finding significant enrichments in brain-related biological processes, such as axonogenesis, and cellular components like the synaptic membrane (fig. 3*a*). We also found enrichments related to the microtubule structure, essential for neural axons and dendrites, consistently present across biological processes, molecular functions, and cellular components (supplementary table S4, Supplementary Material online). This suggests the functional relevance of our identified genes.

We then utilized FUMA (Watanabe et al. 2017) to assess whether BA genes were enriched in upregulated or downregulated DEGs in both brain and nonbrain tissues (see Materials and Methods). Our findings revealed that BA genes were significantly enriched in upregulated DEGs in brain tissues and were generally enriched in downregulated DEGs in nonbrain tissues (fig. 3*b*).

We used GTEx gene expression data (GTEx Consortium 2020), accessed via FUMA online, to compare the expression values of each identified gene between brain and nonbrain tissues. Based on their significance, the identified genes were categorized into three classes: significantly upregulated, significantly downregulated, or insignificant in brain-related tissues when compared with nonbrain tissues (fig. 3*c*; supplementary table S3, Supplementary Material online; see Materials and Methods). Roughly a third of the identified genes, significantly upregulated or downregulated in brain tissues, could potentially contribute to the formation of brain-specific asymmetry features. In contrast, the remaining genes may have universal roles in asymmetry across both brain and nonbrain tissues.

### Evolutionary Origin of BA Genes

**Fig. 4.**
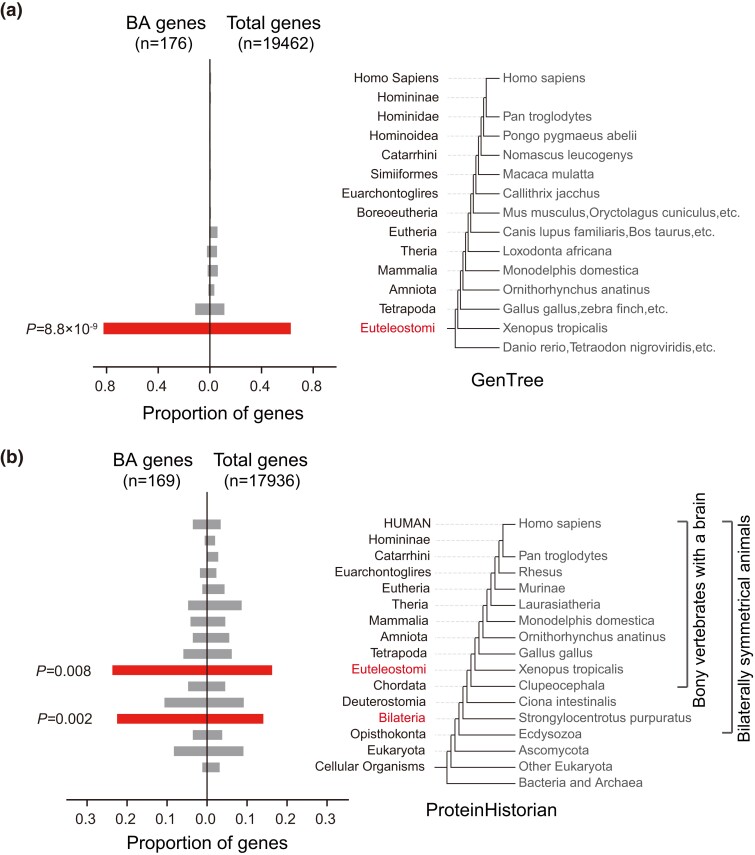
Tracing evolutionary origins of BA genes. (*a*) The left bar graph contrasts the proportion of BA genes in each clade with the total genes’ proportion per clade. A few BA genes that do not overlap with total genes are not considered. The 1:1 corresponding clades, derived from the topological life tree (GenTree, Shao et al. 2019), are presented to the right. The red bar indicates statistical significance based on the binomial test with the corresponding *P* value shown. (*b*) Similar to (*a*), except that the ProteinHistorian tree (Capra et al. 2012) is considered. Bilateria and Euteleostomi correspond to two key evolutionary transitions: the emergence of bilaterally symmetrical animals and the formation of the brain.

In a prior study, GenTree (Shao et al. 2019) mapped the origins of human protein-coding genes onto a phylogenetic tree via synteny comparison, delineating the clade-specific origins of genes. For each clade, we calculated the proportion of BA genes and compared these proportions with those of the total background genes. Using a binomial test for each clade, we found that the ancient clade, Euteleostomi, demonstrated significant enrichment for BA genes (fig. 4*a*; supplementary table S5, Supplementary Material online; see Materials and Methods). Since GenTree relies on synteny, which is accurate for recently originated clades but loses resolution for more ancient clades, our findings suggest that genes underlying brain asymmetry are not newly evolved.

To further illustrate the evolutionary origins of BA genes, we used another set of clade-specific genes as a background, constructed based on protein family information from another study (ProteinHistorian; Capra et al. 2012). ProteinHistorian offers improved resolution in ancient clades, albeit with some accuracy compromise compared with GenTree. Our findings indicated that BA genes are significantly enriched in two ancient clades, Bilateria and Euteleostomi (fig. 4*b*; supplementary table S5, Supplementary Material online; see Materials and Methods).

Bilateria is associated with the origin of bilaterally symmetrical animals (Finnerty et al. 2004). Left–right asymmetry only holds significance within the context of bilateral symmetry. Prior research indicates that bilateral asymmetry may offer certain advantages, though not as universally as bilateral symmetry (Genikhovich and Technau 2017; Vallortigara and Rogers 2020). It is plausible that this trade-off between bilateral symmetry and bilateral asymmetry originated in early Bilateria and has been inherited by extant bilateral organisms.

Euteleostomi, also known as bony vertebrates, is the common ancestral clade of Sarcopterygii and Actinopterygii, the former including all tetrapod species and the latter most fish species (reference: the life map in NCBI, https://lifemap-ncbi.univ-lyon1.fr). The Euteleostomi clade is renowned for its evolution of a complex brain structure—comprising the forebrain, midbrain, and hindbrain—hereafter simply referred to as the “brain” (Šestak and Domazet-Loso 2015). This complexity stands in contrast with the more rudimentary brain structures, such as the nerve cord or neural tube, seen in the primitive members of the Chordata clade. The transition from a simpler to a more complex brain structure during the evolution from Chordata to Euteleostomi coincides with the emergence of three significant anatomical features: the spine, jaw, and bones. The spine provides structural support, the jaw enables a wider range of feeding mechanisms and diets, and the bones offer enhanced bodily integrity and protection. These developments, in turn, may have created the conditions for the evolution of the complex brain structure, indicating a nuanced interplay of selective pressures. As earlier studies suggest, a common basic brain architecture is observable among Euteleostomi species (Hibi and Shimizu 2012; Briscoe and Ragsdale 2019; Hain et al. 2022). It is therefore feasible that the genes responsible for brain formation also contribute to brain asymmetry. Collectively, our findings propose that BA genes originated from two significant evolutionary transitions: the emergence of bilaterally symmetrical animals and the formation of the brain.

In addition, we considered several potential factors that might influence the results. First, we amalgamated the ancient clades preceding Tetrapoda in the ProteinHistorian tree into an “ancient” clade and reperformed the enrichment analysis on this degenerated tree. The findings showed consistent enrichment between the simplified ProteinHistorian tree and GenTree (supplementary fig. S4, Supplementary Material online). Second, we compared the significance of each clade between BA genes defined by plan-I or plan-III and those defined by plan-II or plan-IV, revealing significantly positive correlations between nonabsolute and absolute definitions of brain asymmetry (supplementary fig. S5, Supplementary Material online). Third, we analyzed clade enrichments solely for significantly upregulated and downregulated genes in brain tissues as defined in figure 3*c*, which demonstrated similar enrichments in ancient clades (supplementary fig. S6, Supplementary Material online; see Materials and Methods). Lastly, we considered the nonindependence between statistical tests for different clades and implemented a stepwise statistical test procedure. The results still showed ancient enrichments for both trees (supplementary fig. S7, Supplementary Material online; see Materials and Methods).

In conclusion, the majority of BA genes originate from ancient clades, specifically Bilateria and Euteleostomi, where they are significantly enriched, while a minor portion are of more recent origin as detailed in supplementary table S5, Supplementary Material online.

### Evolution of Gene Expression Underlying Human Brain Asymmetry

**Fig. 5.**
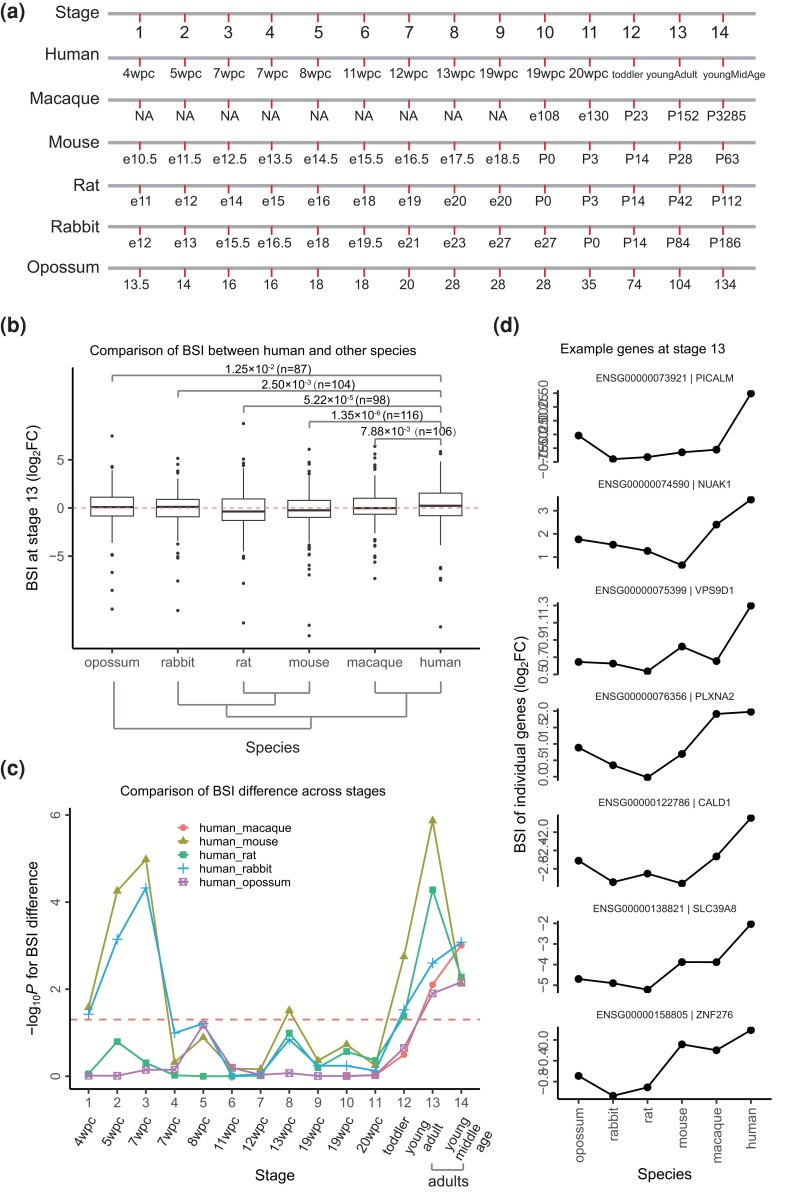
Comparative analysis of BA genes’ BSI across multiple species. (*a*) The diagram displays the corresponding relationships across the developmental stages of different species. The term “NA” denotes the unavailability of data for a specific stage. (*b*) Pairwise boxplots illustrate the comparison of the BSI for BA genes between humans and each of the five other species at stage 13, corresponding to the young adult stage in humans. Below, the species tree is represented topologically. The *P* values of each pair, calculated using a one-tailed pairwise Wilcoxon test, are presented. The red dashed line indicates a BSI of zero. The BSI for each gene is computed as the log_2_-transformed FC between its brain expression and its average expression in other organs (here, the brain vs. the heart, liver, and testis for each species pair), denoted as log_2_(FC). (*c*) This panel displays the comparative analysis of the overall BSI of BA genes between humans and each of the other species across all 14 developmental stages. The *P* values, calculated using a one-tailed pairwise Wilcoxon test, are shown in logarithmic scale. The abbreviation “wpc” refers to weeks postconception. At each stage, a comparison is conducted between humans and each of the other species, wherein the BSIs for both species are calculated based on the organs they have in common. (*d*) This section presents examples of genes that exhibit higher BSI in humans during stage 13.

Considering the relatively recent evolution of most brain asymmetry traits, we are particularly interested in understanding how ancient genes shape these asymmetric characteristics in the human brain. We propose that these genes have gradually developed new functions over time. To investigate this hypothesis, we compiled gene expression profiles from six organs (brain, liver, heart, kidney, ovary, and testis) across six species (human, macaque, mouse, rat, rabbit, and opossum) as reported in a prior study (Cardoso-Moreira et al. 2019). This research assessed gene expressions in these organs at 14 distinct developmental stages, which were harmonized and made comparable across species by integrating developmental transcriptome data and referring to the 23 Carnegie stages that segment embryonic development (Cardoso-Moreira et al. 2019). The corresponding stage relationships are depicted in figure 5*a*. We noted that certain stages in one species may correspond to multiple stages in others; for instance, stage 7 weeks postconception (wpc) in humans matches two stages in mice, rats, and rabbits (fig. 5*a*). Additionally, it is important to mention that data for stages 1–9 in macaques are incomplete (fig. 5*a*; see Materials and Methods).

Orthologous information was obtained from the Ensembl database, and we retained any human gene that has a unique ortholog in at least one of the other five species for further analysis. This process yielded orthologous information for 172 out of the 216 identified genes across the six species. However, we excluded 14 genes specifically associated with cerebellum-related traits, thereby leaving 158 genes for further analysis, as the cerebellum was considered a distinct organ in the original study (Cardoso-Moreira et al. 2019) (see Materials and Methods).

We introduced the Brain Specificity Index (BSI) of a gene, defined as the log_2_ of the fold change (FC) between the gene’s expression in the brain and the average gene expression in other organs (see Materials and Methods). The BSI was calculated for each of the 158 BA genes across the six species and at each of the 14 developmental stages (supplementary table S6, Supplementary Material online; see Materials and Methods). When conducting a comparison for a specific gene between two species, we selected common organs devoid of missing data and with an expression level satisfying the Reads Per Kilobase Million (RPKM) > 1 criterion for the calculation of the BSI. We then established the significance of the BSI in humans being typically larger than in any of the other species using a one-tailed pairwise Wilcoxon test (supplementary table S6, Supplementary Material online; see Materials and Methods). We assessed the robustness of the RPKM > 1 criterion by comparing it with a lower threshold (RPKM > 0.5) and a higher one (RPKM > 1.5), observing a reproducible pattern across these criteria (supplementary fig. S8, Supplementary Material online). Notably, stages 13 and 14, representing young adult and young middle age, respectively, closely correspond to the age range (40–69 years) in which brain traits are typically measured in the UK Biobank.

Initially, we focused on stage 13, which correlates with the young adult stage in humans. The overall BSI of BA genes is significantly higher in humans compared with each of the other species (fig. 5*b*; see Materials and Methods), suggesting a brain-specific upregulation in humans relative to other species. We then investigated when the brain-specific upregulation of BA genes in humans manifests during development, from 4 weeks postconception to young middle age. Our results indicate that the brain-specific upregulation of these genes in humans is gradually established during development and becomes consistently significant in adults (fig. 5*c*; see Materials and Methods). Sporadic signals were detected at 4–7 weeks postconception, 13 weeks postconception, and during the toddler stage when comparing humans with mice, rabbits, and rats (fig. 5*c*). Previous studies on brain asymmetry features in infants have shown that certain traits gradually develop from infancy to adulthood, such as the brain volumes of subcortical regions (Dean et al. 2018). Additionally, specific brain asymmetry traits remain consistent from infancy to adulthood, as observed in the four features within perisylvian regions (Glasel et al. 2011). The early establishment of brain asymmetry is thought to contribute to the development of cognitive competencies (Dehaene-Lambertz and Spelke 2015), suggesting a potential evolutionary pressure (Glasel et al. 2011).

We present examples of genes exhibiting higher BSI in humans at stage 13 (fig. 5*d*). Notably, at stage 13, gene expression profiles are available for four organs in humans: the brain, heart, liver, and testis. These organs are also profiled in the other five species, making stage 13 more suitable for comparison than stage 14, where the number of organs varies between different species pairs. Integrating the results of enrichment analysis for BA genes in upregulated DEGs in brain-related tissues (fig. 3*b*), our comparative analysis of BSI between humans and other species collectively supports the proposition that BA genes have acquired new functions over time.

## Discussion

In this study, we cataloged approximately 1,500 brain asymmetry traits across nearly 100 primary brain regions, representing the most comprehensive investigation into brain asymmetry to date, to the best of our knowledge. We systematically identified the genes associated with these traits, tracing their origins back to two crucial evolutionary transitions: the emergence of bilaterally symmetrical animals and the formation of the brain. Furthermore, we evaluated the likelihood of these ancient genes evolving new functions to drive brain asymmetry by analyzing their evolution in gene expression.

Our research covered a wide range of brain asymmetry features, identifying the most extensive set of BA genes to date. In contrast, a previous study examining 73 brain asymmetry traits identified only a quarter of our total, 52 genes (supplementary table S5, Supplementary Material online) (Sha, Schijven, et al. 2021). When we applied our macroevolution analysis to these 52 genes using ProteinHistorian, we found no significant enrichments in the two ancient clades we identified (supplementary fig. S9*a*, Supplementary Material online). This discrepancy could be due to the limited trait sampling and gene identification in Sha's study compared with our study. Brain asymmetry can be categorized into two types: torque asymmetry, involving asymmetry in 3D space (Li et al. 2018), and asymmetry within corresponding brain regions. We focused on the latter due to its strong association with advanced neural functions. A previous study investigating torque asymmetry identified 68 genes linked to its components (supplementary table S5, Supplementary Material online) (Zhao et al. 2022). When analyzing these genes using ProteinHistorian, we discovered subtle yet significant enrichments in the two ancient clades identified (supplementary fig. S9*a*, Supplementary Material online). Notably, the gene sets from Sha's study and Zhao's study overlap significantly with ours (supplementary fig. S9*b*, Supplementary Material online) and show enrichments in the same ancient clade of GenTree (supplementary fig. S9*c*, Supplementary Material online). By integrating these genes into our study, we found consistent enrichment results (supplementary fig. S9*a* and *c*, Supplementary Material online), reinforcing that both types of brain asymmetries share roots in the same ancient clades. Our larger gene set showed more pronounced significance (supplementary fig. S9*d*, Supplementary Material online).

We also explored asymmetrical gene expression between the left and right brain hemispheres. Our aim was to determine whether the BA genes identified in this study exhibit a higher degree of asymmetrical expression than other genes. Using RNA-Seq data of two donors derived from the Allen Brain Atlas (http://human.brain-map.org/static/download), we computed the asymmetry levels (ALs) for each gene across around 30 paired brain regions for each donor (supplementary table S7, Supplementary Material online; see Materials and Methods). We also averaged these calculations from two donors for their common brain regions considering the subtle yet statistically significant correlation between them (*R* = 0.036, *P* < 2.2 × 10^−16^; see Materials and Methods). We did not observe a significant increase in ALs for the BA genes compared with other genes (*P* = 0.91 for donor 1, *P* = 1.00 for donor 2, and *P* = 1.00 for their average, one-tailed Wilcoxon test). Additionally, we evaluated each brain region separately and identified two regions for donor 1, one region for donor 2, and one region for their average with a *P* value less than 0.05, albeit these did not pass multiple testing (see Materials and Methods). Our results align with a previous study that examined three cortical regions associated with handedness and language in humans and their counterparts in rhesus macaques. This study found that gene expression profiles were generally similar across both hemispheres, except for the human posterior superior temporal cortex that displayed a distinct left–right difference (Muntané et al. 2017). Another study highlighted differential microRNA expression between the left and right hemispheres, suggesting a potential regulatory role of microRNA in establishing brain asymmetry (Miao et al. 2020). Several factors might have contributed to the lack of a significant increase in ALs for BA genes. These include no overlap or sparse sampling in brain regions, asymmetry traits being contributed by specific cells rather than all cells, timing mismatches between asymmetry trait determination and gene expression measurement, or the limited availability of gene expression data from only two individuals.

The origins of brain structural asymmetry have long fascinated scientists. Identifying conserved brain regions and structural asymmetries is a crucial step in decoding the evolutionary process. While some asymmetries are found in model organisms like mice (Barbeito-Andres et al. 2016) and zebrafish (Duboc et al. 2015), many are unique to humans or primates, distinguishing them from other species. Challenges in identifying these conserved features across various species hinder progress (Leroy et al. 2015; Marie et al. 2018; Graic et al. 2020; Neubauer et al. 2020; Xiang et al. 2020; Wan et al. 2022). Uncovering the BA genes could provide insights into the evolutionary mechanisms underlying brain structural asymmetry, informing several crucial areas of research. For instance, BA genes could serve as markers for determining corresponding brain regions across various species. Researchers can also explore how BA genes acquire new functions by examining the variants in their gene sequences, along with their regulatory sequences. It would be worth investigating whether the asymmetrical contribution of BA genes to brain asymmetry during development could be spatial (asymmetrical gene expression between two hemispheres), temporal (asynchronized gene expression between two hemispheres during development) (Palomero-Gallagher and Amunts 2022), or both. Understanding how this asymmetry develops prematurely or belatedly in the developmental process and its contribution to evolutionary pressure presents a worthy challenge. Such an understanding could prove beneficial for studies related to associated diseases.

Symmetry in animals often signifies developmental robustness, underpins the “good genes” hypothesis of sexual selection (Byers and Waits 2006), and influences art and aesthetics (Bertamini et al. 2019). However, asymmetry is also observed in nature, as exemplified by the claws of fiddler crabs, the eyes of flatfish, and internal organs like the heart, suggesting adaptive benefits in certain contexts (Duboc et al. 2015). Among these, brain asymmetry is particularly intriguing due to its connection to functional brain divisions and its proposed role in the evolution of intelligence (Wu et al. 2022). Various theoretical models have been proposed to explain specific facets of brain asymmetry (Ocklenburg and Gunturkun 2022). These include the dextral/chance model for hand preference and language dominance (McManus 1985), the pathological left-handedness model (Satz et al. 1985), the right-ear advantage model for left-hemisphere dominance in speech and language (Godfrey and Grimshaw 2016), and the valence lateralization model for emotional lateralization (Palomero-Gallagher and Amunts 2022). Among these theories, the Geschwind–Galaburda–Behan model holds considerable significance. This model proposes that elevated fetal testosterone levels can retard left hemisphere development, potentially resulting in right-hemisphere dominance, left-handedness, immune disorders, and dyslexia (Geschwind and Galaburda 1985). Remarkably, among the BA genes we identified in our study, *KANSL1* stands out—a gene previously reported to underlie dyslexia (Paracchini et al. 2016). Manns’ (2021) multilevel model postulates that genetic factors instigate basic brain asymmetry during embryonic development, which is then sculpted by functional needs, subsystem interactions, and environmental influences. This theory harmonizes with the gradual establishment of the brain-specific upregulation of BA genes during development. Additionally, the “from hand to mouth” theory presents an intriguing connection between primate gestural communication and analogous human language regions (Corballis 2002). Empirical cross-species studies have significantly enriched this field. For instance, mustached bats demonstrate a leftward asymmetry for social vocalizations in the primary auditory cortex, similar to humans (Washington et al. 2021). Also, larger-brained bird species have been reported to show stronger foot preferences, analogous to human hand preferences (Kaplan and Rogers 2021). Importantly, the BA genes we identified in our study could serve as molecular markers, providing a foundational basis for these theoretical models and offering evolutionarily conserved molecular evidence for empirical studies.

Despite the significant insights, our study has some limitations. These include the need for further investigation into the evolutionary mechanisms of BA genes and the mechanisms driving their upregulation. There is also the challenge of exploring the interplay between BA genes and environmental factors impacting brain asymmetry. Among the BA genes we identified, several are of recent origin (supplementary table S5, Supplementary Material online) and, despite not displaying a significantly higher ratio than expected, they demand further study due to their potential contributions to brain asymmetry. Our gene identification method could be enhanced through the use of advanced annotation algorithms, fine mapping, and experimental validation. Future studies would benefit from larger sample sizes and ethnically diverse populations to improve the generalizability of our findings.

## Materials and Methods

### Genotype Preprocessing in UK Biobank

The UK Biobank database received ethical approval from the National Research Ethics Service Committee North West-Haydock (reference 11/NW/0382), and all procedures adhered to the World Medical Association Guidelines. All participants provided informed consent. The cohort comprises individuals aged between 40 and 69 years, primarily of White British ancestry, with a minority representing other ancestries. Approximately 50,000 participants were analyzed using the Applied Biosystems UK BiLEVE Axiom Array by Affymetrix, while the remaining around 450,000 participants were run on the Applied Biosystems UK Biobank Axiom Array, which shares 95% marker content with the former (Bycroft et al. 2018).

The genotype data we downloaded comprised 93,095,623 imputed SNPs for 487,411 individuals. We conducted the following preprocessing steps on the genotype data: initially, we excluded SNPs with a minor allele frequency (MAF) of less than 0.01 and an imputation INFO score of less than 0.8. Subsequently, we retained SNPs with a genotype call probability greater than 0.9 using the qctool. We also ensured that only biallelic SNPs were included. Finally, we excluded SNPs with a *P* value of less than 10^−6^ based on Hardy–Weinberg equilibrium testing and those with a genotype missing rate greater than 0.05 for both White and non-White British populations.

### Definition and Preprocessing of Brain Asymmetry Trait

Traits within the UK Biobank are designated by field number, instance number, and array number, formatted as “field number-instance number.array number.” For instance, the trait coded as “25565-2.0” pertains to field 25565, measured in instance 2, with an array number of 0. In this study, we collected data for all brain-related traits measured in paired brain regions (left and right hemispheres). Each trait contains two instances: instance 2, representing the imaging visit (2014+), and instance 3, denoting the first repeat imaging visit (2019+). These instances were treated as two separate time periods, aiding in evaluating the impact of temporal variation on the identification of genetic variants. All traits analyzed in this study had an array number of 0.

Using “*L*” to signify a trait measuring a specific brain region in the left hemisphere and “*R*” to denote a trait measuring the corresponding region in the right hemisphere, we defined brain asymmetry traits for each pair of these traits by $arcsin((L-R)/\sqrt{2(L^{2}+R^{2})})$ or $arcsin((L_{s}-R_{s})/\sqrt{2(L_{s}^{2}+R_{s}^{2})})$, as well as their absolute form, where “*L*_s_” and “*R*_s_” denote the standardized values of the traits “*L*” and “*R*,” respectively. As a result, we generated four asymmetry traits for each trait pair. We subsequently excluded asymmetry traits with a sample size of fewer than 1,000, as well as those traits where more than 50% of the participants from the White British population shared identical values. We carried out normalization transformations for each of these asymmetry traits, considering White and non-White British populations separately. The primary normalization was performed using the R function “bestNormalize”; in cases where poor transformation was obtained, we substituted this function with “scale.”

A thorough mathematical review of the advantages of our revised definitions over the traditional formula and their connections is detailed in the supplementary note, Supplementary Material online.

### QTL Mapping

Our analysis first involved excluding subjects with discrepancies between their self-reported (Field 31) and genetically inferred sex (Field 22001). We then applied linear regression to remove the influence of covariates, including age (Field 31), age^2^, age × sex, age^2^ × sex, and the top 20 principal components (Field 22009-0.1 to 22009-0.20) that capture population genetic diversity. The residuals obtained were used for subsequent QTL mapping.

We computed the genetic relatedness matrix (GRM) using the overlapping SNPs with LD-pruned HapMap3 SNPs for White and non-White British populations, in accordance with a previous study (Jiang et al. 2019). The LD pruning process was executed via PLINK (parameters: --indep-pairwise, window size = 1000 variant count, step size = 100 variant count, pairwise r2 = 0.9 and MAF = 0.01). Thereafter, a sparse GRM was generated with GCTA (--make-bK-sparse 0.05). We then undertook QTL mapping using GCTA (--fastGWA-mlm) with the sparse GRM as an input, resulting in summary statistics for each asymmetry trait.

We determined significant SNPs at a threshold of *P* < 5 × 10^−8^, removing SNPs with a MAF of less than 0.01. Finally, PLINK was used to perform clumping analysis (r2 = 0.5 and kb radius = 250). Postclumping, we obtained the lead SNPs or index SNPs, each representing a QTL.

### Replication Analysis

We defined three phases as follows: Phase 1 comprises asymmetry traits measured during the first time period for the White British group, identified by Field 22006, and includes approximately 30,000 individuals with 8,895,704 SNPs. Phase 2 encompasses asymmetry traits measured during the same period but for the non-White British group. Phase 3 consists of asymmetry traits measured during the second time period for the White British group, which includes around 1,000 individuals. Phases 2 and 3 are subsequently used as replication data sets.

Determining the non-White British group involved three steps. First, we identified individuals who reported as non-White in Field 22006. Second, we estimated the GRM for this group, obtained the sparse GRM, and removed subjects exhibiting relatedness with more than 100 other individuals in the sparse GRM. These individuals were identified as having mixed genetic backgrounds. Finally, subjects reporting mixed genetic backgrounds or those with missing values in Field 21000 (21000-0.0) were excluded. This second step was repeated to ensure a relatively clear genetic background. As a result, the non-White British group comprised approximately 3,000 individuals.

To evaluate the consistency between the discovery (phase 1) and replication (phases 2 and 3) data sets for traits with QTLs in the discovery data set, we utilized three indexes. First, we calculated Pearson’s correlation (*R*) between the *β* coefficients of the discovery data set and those of a replication data set for each trait, using the top 200 SNPs obtained through clumping in the discovery data set (--clump-p1 0.0001 --clump-p2 0.0001). Second, we assessed the SCR between the *β* coefficients of the top 200 SNPs in the discovery data set and a replication data set. The significance was evaluated using a binomial test (expected success probability = 0.5). We combined all *β* coefficients of the top 200 SNPs for traits with QTLs to yield a single SCR. Third, we identified QTLs (lead SNPs) discovered in the replication data set that were initially identified in the discovery data set. The significance of this identification was evaluated using a binomial test with an expected success probability equal to 0.05/*n* (Bonferroni correction), where “*n*” is the number of overlapping lead SNPs in a replication data set.

### Gene Annotation

To annotate the associated genes for each of the lead or index SNPs, we utilized both ANNOVAR and PhenoScanner. ANNOVAR accepts SNP locations as input, while PhenoScanner uses SNP identifiers. For the annotations provided by ANNOVAR, we eliminated instances of multiple-gene annotation for a single SNP, including intergenic annotations. Using the Ensembl ID annotation information provided by the HGNC database (version 2023-3-2), we translated the gene names given by ANNOVAR into Ensembl IDs. As PhenoScanner provides a unique Ensembl annotation for each SNP, we directly collected the Ensembl IDs it offered. Ultimately, we combined the annotations from both software solutions due to the high degree of overlap in their results.

### Functional Enrichment Analysis

To perform GO enrichment analysis, we employed the R package “clusterProfiler,” inputting Ensembl IDs and referencing “org.Hs,.eg.,db.” Subsequently, we utilized FUMA's online platform to conduct tissue enrichment analyses, adhering to default settings. The GO enrichment analysis underscored significant gene enrichments in biological processes, cellular components, and molecular functions. FUMA's tissue enrichment analysis revealed considerable enrichments of BA genes in both upregulated and downregulated DEGs across various tissues. We identified significantly upregulated or downregulated genes in brain tissues compared with nonbrain tissues using a two-tailed *t*-test, with a significance level of 0.05, adjusted for multiple comparisons with Bonferroni correction. Notably, of the 216 genes, 16 were not present in the results generated by FUMA.

### Inferring Gene Origin by Macroevolution Analysis

We referenced two previous studies, GenTree and ProteinHistorian, which identified clade-specific origins of genes. Each of these studies features key evolutionary clades, with genes that emerged at each clade mapped by the original authors through synteny comparison and protein family analysis, respectively. We contrasted the distribution of the identified BA genes across these clades against the total gene distribution as a baseline. A binomial test was employed to gauge the significance of enrichment at each clade, setting a significance level at 0.05. This procedure was replicated, considering only genes significantly up- or downregulated in brain tissues. For comparison between ProteinHistorian’s results and those from GenTree, we grouped all clades preceding Tetrapoda into an “ancient” clade within ProteinHistorian. To address potential nonindependence issues between evaluations of different clades, we conducted a stepwise binomial test. For instance, in using ProteinHistorian, we first identified Bilateria as the most significant clade. We then removed the genes at clade Bilateria from both the BA gene set and the background gene set. Subsequently, we applied the same binomial test to the remaining clades to identify the most significant among them. This process was reiterated until no additional significant clades were detected. Of the 216 BA genes we identified, 176 overlap with the total background genes in GenTree, while 169 do so in ProteinHistorian.

### BSI of Gene Expression

Given a developmental stage and a specific species, the expression levels of a gene in six distinct organs (brain, heart, liver, kidney, ovary, and testis) of that species are represented as a vector ($\bar{x_{1}},\bar{x_{2}},\bar{x_{3}},\bar{x_{4}},\bar{x_{5}},\bar{x_{6}}$). Each element of this vector corresponds to one of these organs. The BSI of this gene is computed as BSI = $\log_{2}\;\left(\frac{\bar{x_{1}}}{\bar{\sum_{i=2}^{6}\bar{x_{i}}}}\right)$, where $\bar{x_{i}}$ represents the average gene expression of the focal gene in the *i*th organ among the biological replicates of this organ, $\bar{x_{1}}$ represents that in the first organ, that is, the brain, and $\bar{\sum_{i=2}^{6}\bar{x_{i}}}$ denotes the average expression level in the other organs.

To assess whether BA genes exhibit a significantly higher BSI in humans than in another species, we first kept the common organs without missing values and with expression level satisfying the criterion RPKM > 1 in both species, and then we employed a one-tailed pairwise Wilcoxon test to determine the *P* value. The robustness of the criterion is evaluated by comparing the results of it with those of another two criteria, RPKM > 0.5 and RPKM > 1.5. The analysis excludes the red junglefowl from the original study (Cardoso-Moreira et al. 2019) due to the unavailability of its Ensembl IDs from the Ensembl database. The cerebellum is not considered in the analysis as it was treated as an independent organ in the original study, and only a small fraction of our investigated traits relates to it.

At stage 13, gene expression profiles are available for four organs in humans: the brain, heart, liver, and testis. These organs are also profiled in the other five species, making stage 13 more suitable for comparison than stage 14, where the number of organs varies between different species pairs. Due to the consistent number of organs at stage 13, the BSI for most genes can be calculated consistently across all species, with only a few exceptions. Therefore, when creating the plots in figure 5, we used the average BSI of genes obtained from comparisons between humans and each of the other species. Notably, we calculated the *P* values based on the BSI, which was determined using the common organs for each gene between species pairs. This approach ensures a rigorous comparison.

### Comparing Asymmetry in Gene Expression: BA Genes Versus Others

We obtained RNA-Seq data of two donors from the Allen Brain Atlas (http://human.brain-map.org/static/download), which includes measurements for approximately 20,000 genes across around 30 brain regions (32 for donor 1 and 34 for donor 2) in both the left and right hemispheres. These brain regions were identified using the annotation files that were downloaded with the RNA-Seq data. We then calculated the AL of gene expression between the left and right hemispheres for each brain region, using the formula $|L-R|/\sqrt{2(L^{2}+R^{2})}$. This calculation is consistent with the plan-II of our asymmetry definitions, although it does not include an arcsine transformation. Theoretically, based on this definition, the AL could range from 0 to 1.

When multiple measurements were made for a left or right brain region, we used the average expression of these replicates to provide a single gene expression value, omitting any missing values. If a gene had missing values in either hemisphere for a specific pair of brain regions, the AL for that gene between those regions was also reported as a missing value. To minimize bias from low expression values, we classified expression values in the original data with a relative TPM less than 1e^−7^ as missing values. This accounted for 22% of the data for donor 1 and 21% for donor 2. Consequently, there remain 14 regions for donor 1 and 12 regions for donor 2 where nonmissing AL values were obtained.

After conducting these calculations, we obtained the AL for BA genes and other genes across all pairs of brain regions. We used a one-tailed Wilcoxon test to determine whether BA genes had a significantly higher AL than other genes, yielding the corresponding *P* value. A total of 174 BA genes were subjected to this AL analysis. The gene identities were determined by first transforming their Ensembl IDs into Entrez IDs, using the annotation information provided by the HGNC database (version 2023-3-2) and then aligning them with the Entrez IDs measured in the Allen Brain Atlas. Notably, the brain regions measured in the Allen Brain Atlas represent only a small proportion of total brain regions, based on the provided annotation files.

We measured seven brain regions shared in both donors and found a significant correlation between the ALs for each gene across these regions (Pearson's *R* = 0.036, *P* < 2.2 × 10^−16^). We then averaged the AL for each gene across these regions and conducted the same statistical testing. When we applied this testing individually to each brain region for both donors and their average, we identified two regions for donor 1 (LiG-pest with *P* = 0.0099 and LOrG with *P* = 0.032), one region for donor 2 (ITG-mts with *P* = 0.014), and one region for their average (MTG-s with *P* = 0.028), all with a *P* value less than 0.05. However, these did not survive correction for multiple testing via the Benjamini–Hochberg method. In this context, LiG-pest represents the lingual gyrus, left/right, peristriate; LOrG represents the lateral orbital gyrus; ITG-mts represents the inferior temporal gyrus, left/right, bank of mts; and MTG-s represents the middle temporal gyrus, left/right, superior bank of gyrus.

## Supplementary Material

Supplementary data are available at *Molecular Biology and Evolution* online.

## Supplementary Material

msad181_Supplementary_Data

## Data Availability

Supplementary material and the code used for analysis in this study can be found on GitHub at https://github.com/Jianguo-Wang/BAgenes.
