# Supplementary material for: Evolution of Human Brain Left–Right Asymmetry: Old Genes with New Functions
Source: Mol Biol Evol. 2023 Aug 10;40(9):msad181. doi: 10.1093/molbev/msad181 (PMC10473864; doi:10.1093/molbev/msad181)
Supplement: msad181_Supplementary_Data [file msad181_supplementary_data.zip › SupplementaryMaterial.pdf]

Supplementary Material of

"Evolution of human brain left-right asymmetry -- Old genes with new functions"

Jianguo Wang[1], Sidi Ma[1], Peijie Yu, & Xionglei He

State Key Laboratory of Biocontrol, School of Life Sciences, Sun Yat-sen University, Guangzhou 510275, China

[1] These authors contributed equally

Correspondence should be addressed to:
X.H. (hexiongl@mail.sysu.edu.cn) or J.W. (wangjg22@mail2.sysu.edu.cn)

This file contains:

Supplementary Figs. S1-S10

Supplementary Note

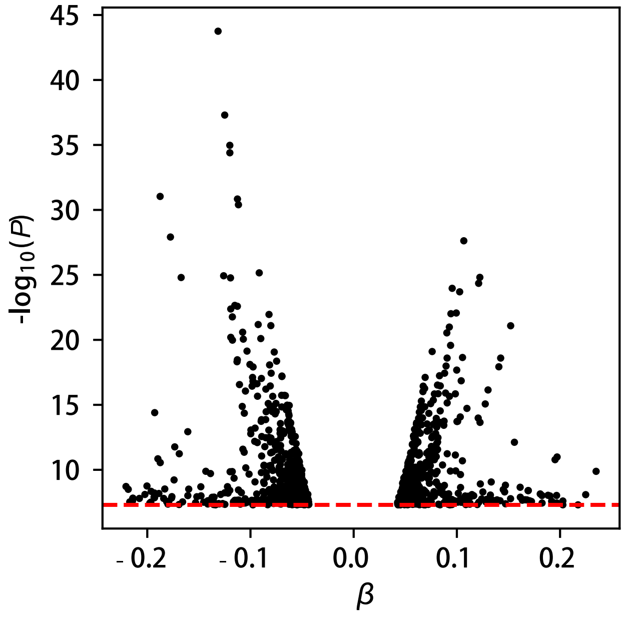

Fig. S1. Representation of Effect Sizes (β) for QTLs Corresponding to Their P-Values.

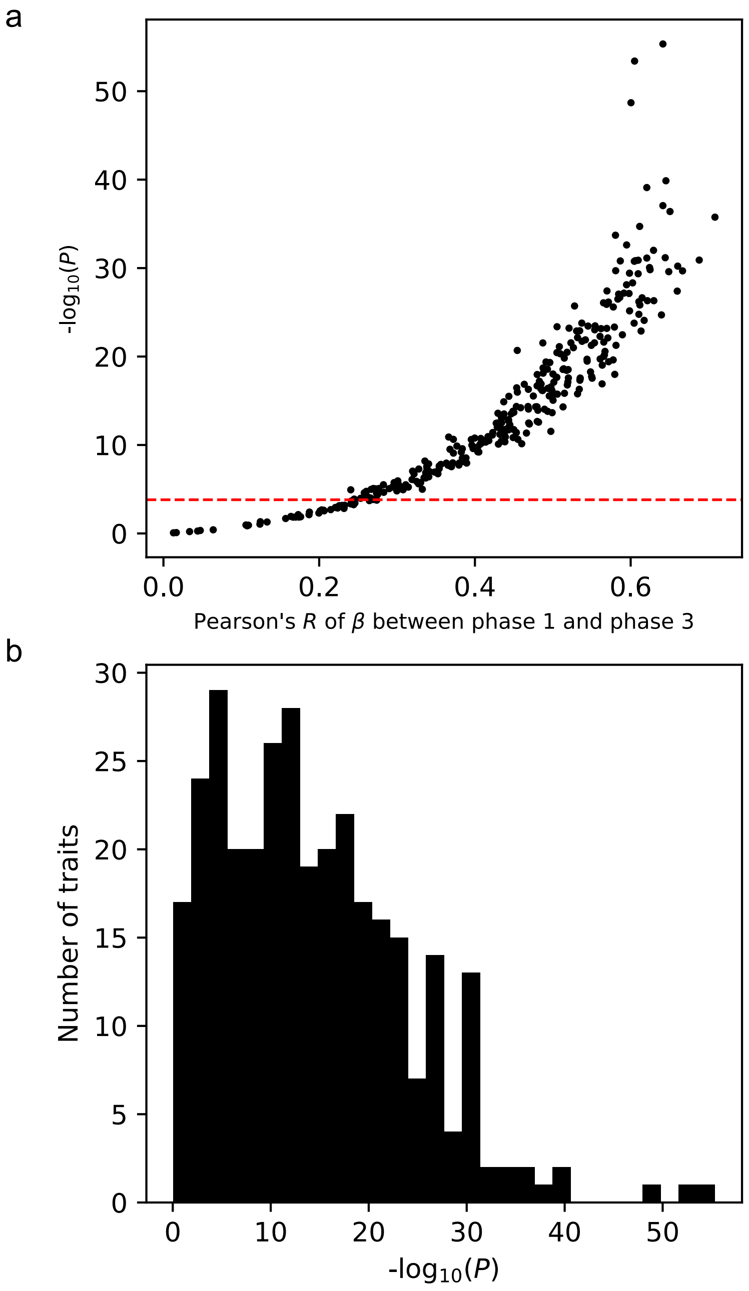

Fig. S2. Pearons's correlation (*R*) between the *β* coefficients of phase 1 and 3. (a) Illustrates the p-values associated with the ***R*** between the ***β***'s of phases 1 and 3 for each trait. **(b)** Depicts the distribution of p-values on a logarithmic scale.

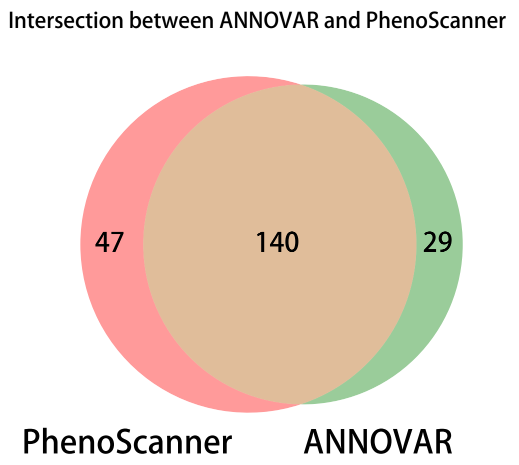

Fig. S3. Overlap of Genes Annotated by ANNOVAR and PhenoScanner.

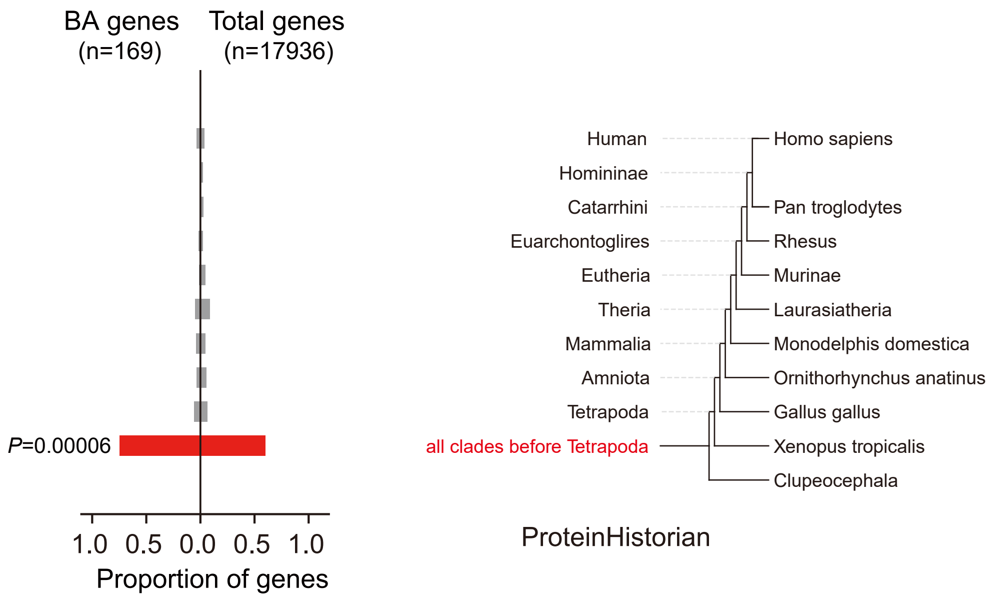

Fig. S4. Inferring the Origin of BA Genes Using a Degenerated ProteinHistorian Tree. All of the clades preceding Tetrapoda are grouped into a single clade, making it comparable to the ancient Euteleostomi clade in GenTree. The red bar denotes statistical significance as determined by the binomial test, with the p-value displayed.

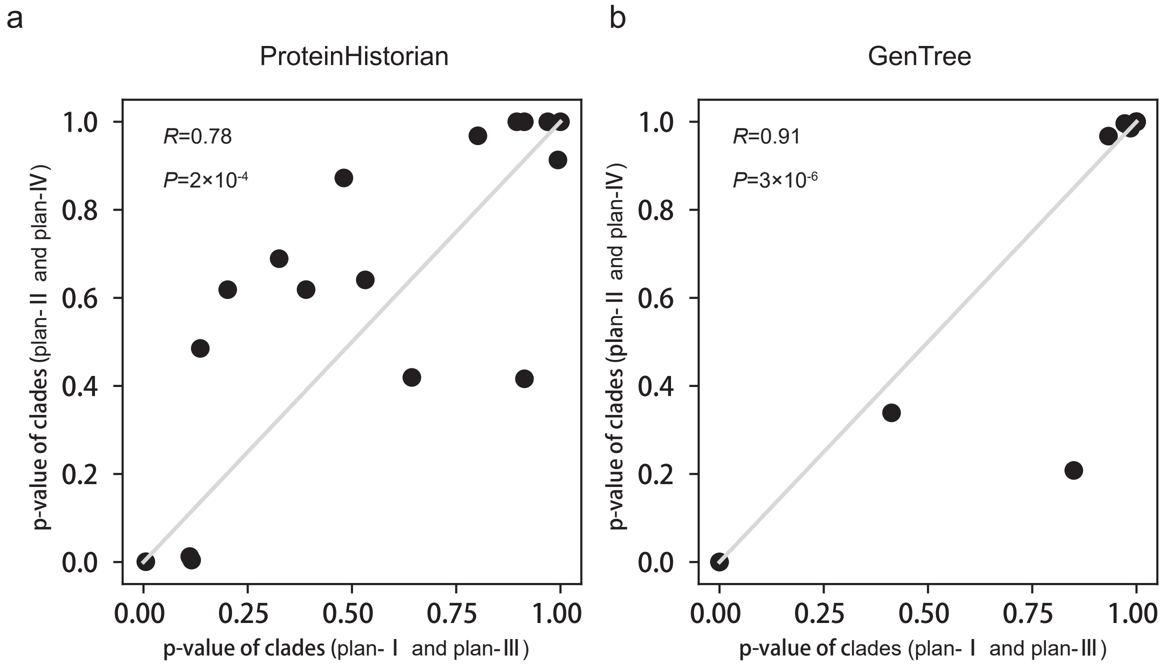

Fig. S5. Comparison of Absolute and Non-Absolute Definitions of Asymmetry Traits. (a) This panel compares the p-values of genes annotated for clade enrichment in the ProteinHistorian tree, based on absolute and non-absolute definitions of brain asymmetry traits. A significant positive correlation is observed. **(b)** This panel is similar to (a) but considers the GenTree instead.

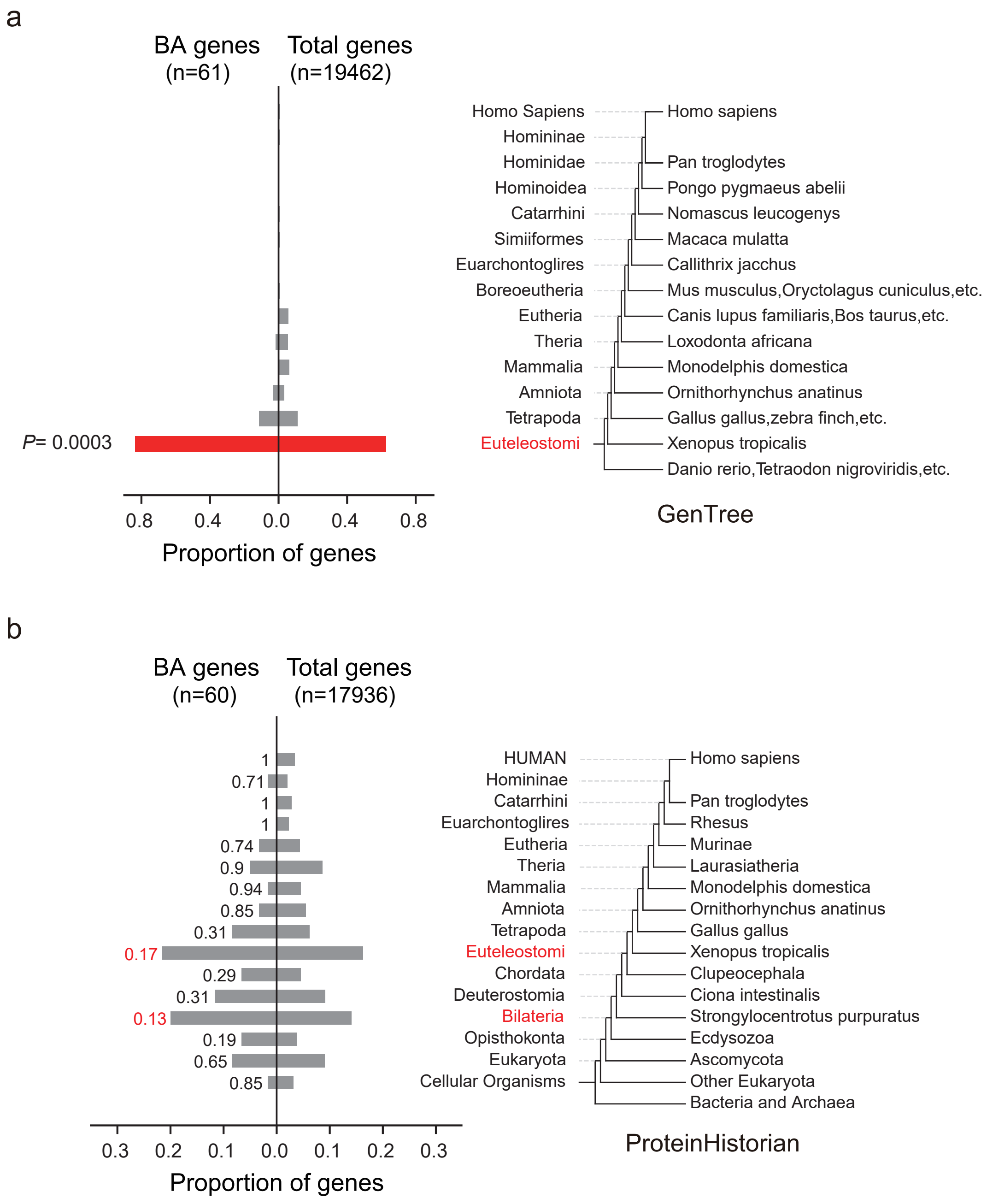

Fig. S6. Gene Origin Analysis for Genes Significantly Up-Regulated or Down-Regulated in Brain Based on Fig. 3c. (a) In GenTree, the ancient clade exhibits significant enrichment. The red bar indicates statistical significance determined via a binomial test, with the corresponding p-value shown. **(b)** In ProteinHistorian, the two clades, Euteleostomi and Bilateria, exhibit the smallest p-values, denoted in red.

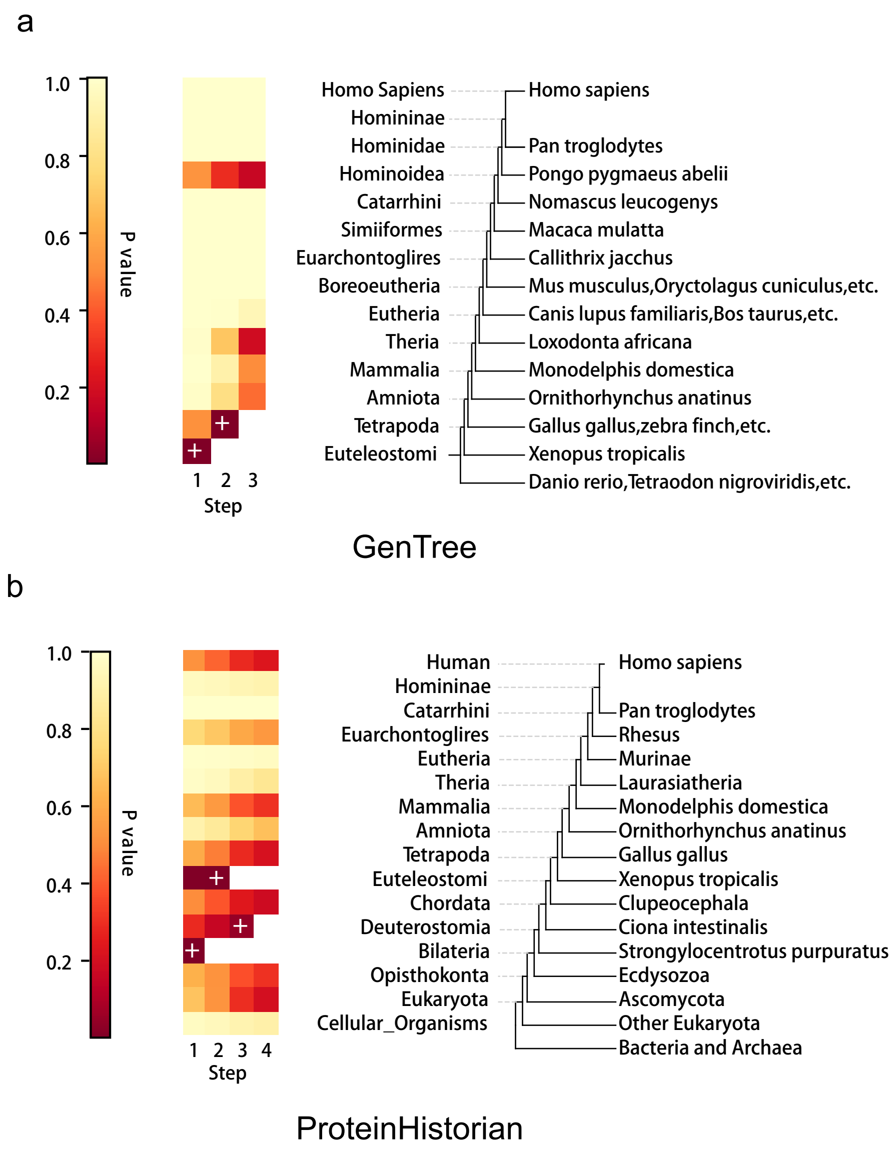

Fig. S7. Stepwise Procedure for Significant Enrichment Testing in Clades. **(a)** In GenTree, the Euteleostomi is identified as the first significant clade. Darker shades represent smaller p-values. The symbol "+" indicates significance at a 0.05 level. **(b)** In ProteinHistorian, the first and second identified significant clades are Bilateria and Euteleostomi, respectively. Darker shades denote smaller p-values, and the "+" symbol denotes significance at a 0.05 level. When comparing with Fig. 4a-b, an additional clade is identified in the vicinity of significant clades.

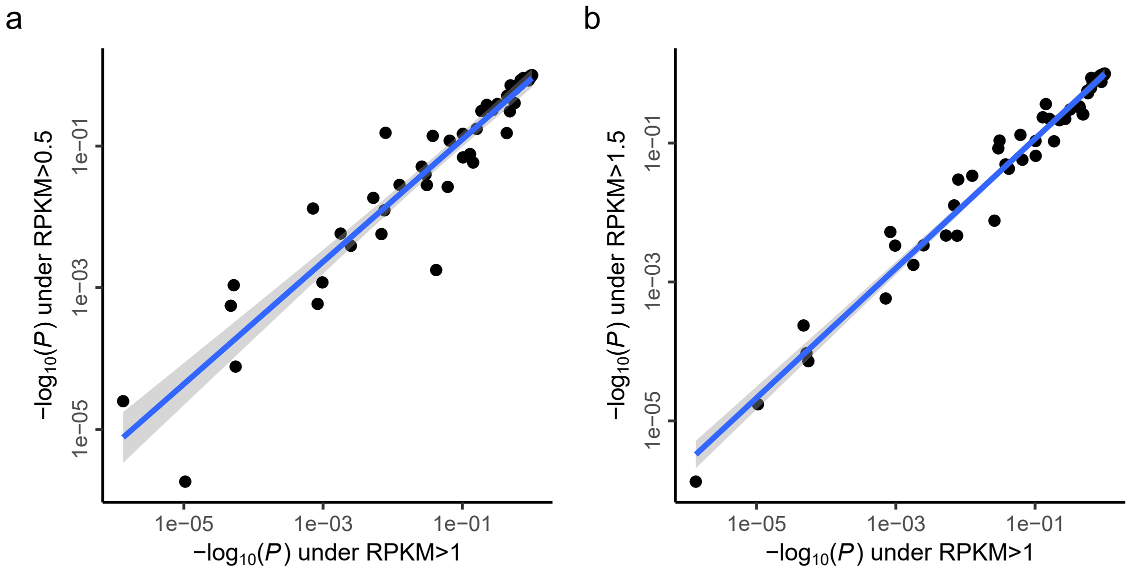

Fig. S8. Evaluating the Robustness of the RPKM Criterion.
In the main text, we utilized a criterion of RPKM>1 to filter out lowly expressed genes. To examine the robustness of this criterion, we adjusted it to two different values, 0.5 and 1.5, and recalculated the BSI for each BA gene and each species. We then determined the p-value to assess whether humans have a significantly higher BSI compared to any other species. **(a-b)** Illustrate the comparison between the original criterion (RPKM>1) and the two adjusted criteria (RPKM>0.5 and RPKM>1.5) respectively, using a logarithmic scale that gives more weight to smaller p-values. The blue line is fitted using linear regression, with the grey band representing the 95% confidence interval. Each point corresponds to a p-value derived from a one-tailed pairwise Wilcoxon test under different RPKM criteria.

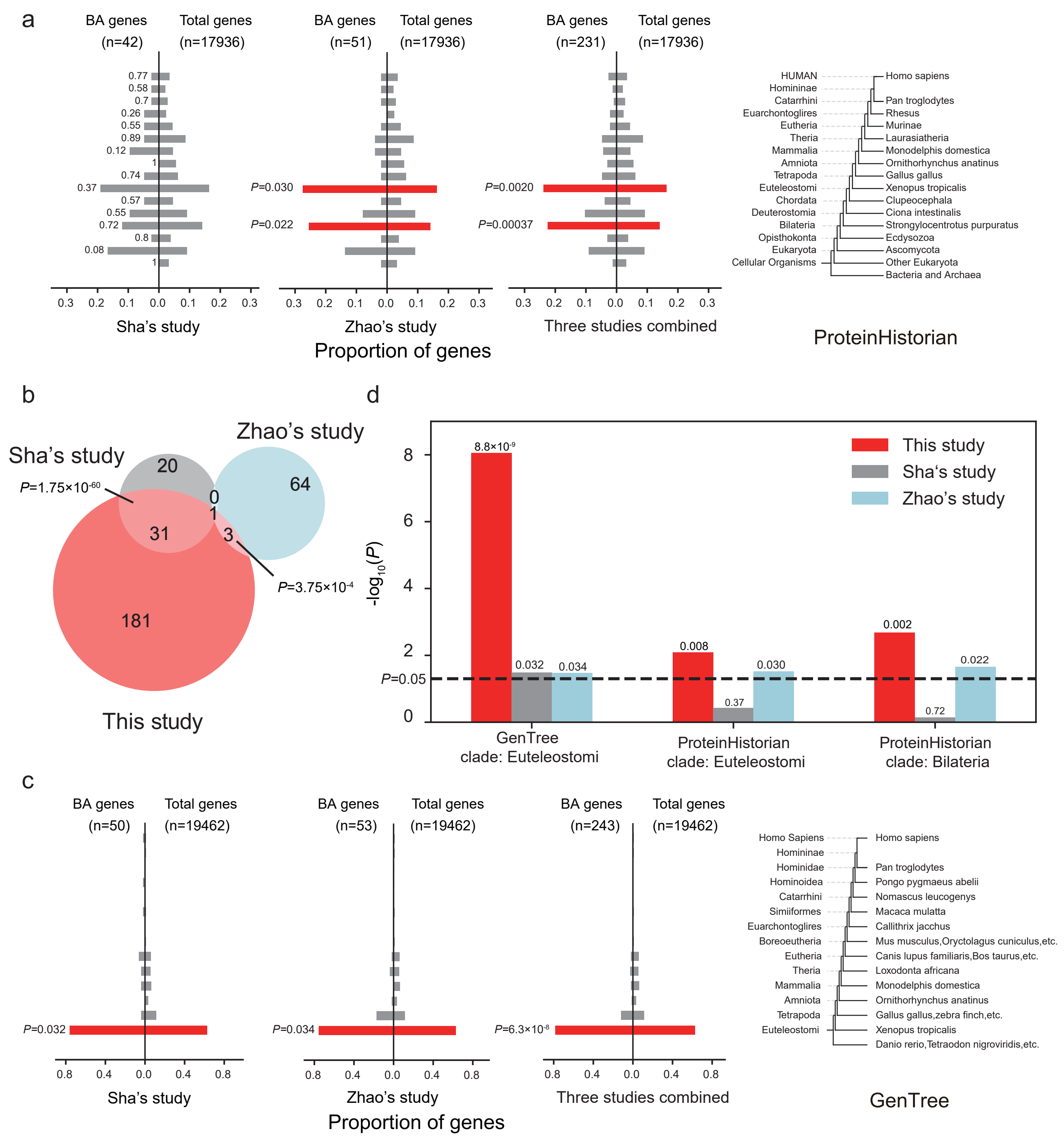

Fig. S9. Comparative Macroevolution Analysis: Our Study vs. Sha and Zhao.
(a) Macroevolution analysis of Brain Asymmetry (BA) gene enrichment from our study, Sha's (left barplot), Zhao's (middle barplot), and a combination of all three (right barplot). Each barplot's left side shows the proportions of BA genes within each ProteinHistorian clade, while the right side provides total gene proportions as a reference, akin to Fig. 4. Clades with significant BA gene enrichments are marked red with corresponding p-values. Absence of significant enrichments prompts the listing of all p-values. The number of BA genes per barplot is indicated in brackets. **(b)** Depicts overlaps between gene sets from any two studies. Our gene set shows significant overlap with Sha's and Zhao's, as validated by binomial test p-values, based on Ensembl IDs. **(c)** Similar to (a), but uses GenTree for gene origin information. **(d)** Compares enrichment significance across three clades, showing significant BA gene enrichment in our study, against those from Sha's and Zhao's studies. P-values are provided with a black dashed line signifying a p-value of 0.05.

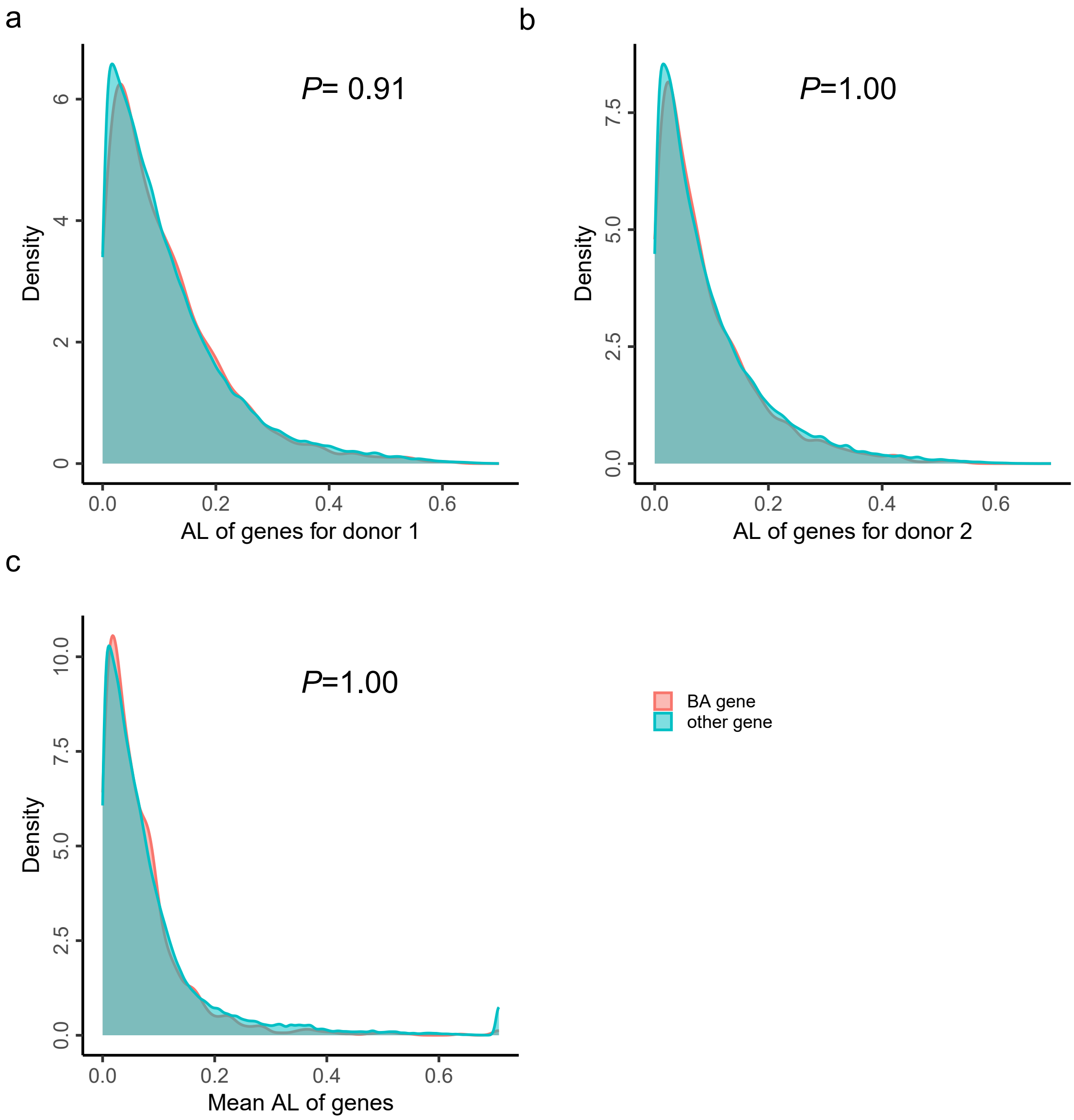

Fig. S10. Comparison of the Asymmetry Level (AL) of BA Genes and Other Genes. In this comparison, 'mean AL' refers to the average AL of two donors. The p-value is calculated using a one-tailed Wilcoxon test. The AL is defined as $|L-R|/\mathrm{Sqrt}(2(L^2+R^2))$, mirroring our definition of brain asymmetry traits, but without implementing the arcsine transformation. Theoretically, the AL ranges from 0 to 1. **(a-c)** Illustrate the AL of genes for donor 1, donor 2 and the average AL of genes across seven shared brain regions.

Supplementary Note

Mathematical Comparison Between Different Definitions of Asymmetry Level (AL)

Bridging Traditional Definition and Our Definition

Firstly, we explore the link between the traditional definition of AL, expressed as AL=(L-R)/(L+R), and our Plan-I definition, formulated as AL=arcsin((L-R)/Sqrt(2*(L^2+R^2))). The traditional definition can be reshaped as follows: AL=(L-R)/(L+R)=1-2R/(L+R)=1-2/(L/R+1), where L/R signifies the cotangent of the deviation angle of the vector (L, R) from the horizontal axis (i.e., R=0). As the angle transitions from 0 to 90°, L/R shifts from +∞ to 0, and AL moves from 1 to -1. Notably, AL is a monotonically decreasing function of the deviation angle of (L, R) from R=0. Nevertheless, the deviation angle used in the traditional definition lacks intuitive clarity since it measures the deviation from R=0 without accounting for L, while we will establish the equivalence of the traditional definition to our Plan-I definition.

Our Plan-I definition provides a more comprehensible geometrical interpretation. As demonstrated in Fig. 1b, it represents the deviation angle from the diagonal line (i.e., L=R). Given that the deviation angle from L=R is 45° minus the deviation angle from R=0 when the point resides in the first quadrant, our definition is a monotonically decreasing function of the deviation angle used in the traditional definition. Thus, our definition is a monotonically increasing function of the traditional definition. When normalization is applied to the derived AL traits, both definitions yield the same distribution. Accordingly, the two definitions are equivalent when both L and R are positive and the derived AL trait is normalized.

Limitations of the Traditional Definition

However, the traditional definition presents two prominent issues. The first is that the traditional definition struggles when L and/or R encompass both positive and negative values. A critical failure arises when $L+R=0$. More crucially, when both positive and negative values are present, the traditional definition may inaccurately assign left-preference or right-preference. For instance, consider the two trait pairs, $(L, R)=(1, 2)$ and $(L, R)=(-1,-2)$. Using the traditional definition, we deduce that both trait pairs are right-preference, whereas in reality, the first pair is right-preference and the second is left-preference since $L<R$ for the first pair and $L>R$ for the second. In contrast, using our definition, we achieve an accurate reverse assignment for left-preference or right-preference.

A second limitation stems from the first. The traditional definition finds difficulty when L and/or R comprise both positive and negative values, complicating the consideration of trait value distribution. Specifically, if both L and R follow a normal distribution, a more appropriate reference point would be the population average rather than the origin, necessitating the scaling of L and R by their mean. Our definition capably handles trait distributions as it measures the deviation angle from the diagonal line, $L=R$. We regard the average as the reference point and develop Plan-III of our four definitions. Plans-II and IV are absolute versions, only considering the asymmetry level and disregarding the asymmetry direction, i.e., left-preference or right-preference.

In conclusion, our definitions consider both the origin and the average as two potential reference points, collectively evaluating whether an asymmetry trait is inherited with or without direction. Notably, our first definition aligns with the traditional one when both L and R are positive and the derived trait is subject to normalization.
